# Research Progress on the Cardiac Injury from ACE2 Targeting in SARS-CoV-2 Infection

**DOI:** 10.3390/biom11020196

**Published:** 2021-01-30

**Authors:** Hao Sun, Xiaojuan Su, Lingyi Huang, Dezhi Mu, Yi Qu

**Affiliations:** 1 Department of Pediatrics/Key Laboratory of Birth Defects and Related Diseases of Women and Children (Ministry of Education), West China Second University Hospital, Sichuan University, Chengdu 610041, China; shuang19830403@163.com (H.S.); xiaojuansu2017@163.com (X.S.); mudz@scu.edu.cn (D.M.); 2 West China College of Stomatology, Sichuan University, Chengdu 610041, China; 2017151642114@stu.scu.edu.cn

**Keywords:** ACE2, COVID-19, SARS-CoV-2, spike

## Abstract

The epidemic due to the novel coronavirus (SARS-CoV-2) is now a global concern, posing a severe threat to the health of populations. At present, all countries in the world are stepping up the development of vaccines and antiviral agents to prevent the infection and further transmission of SARS-CoV-2. An in-depth investigation of the target organs and pathogenesis regarding SARS-CoV-2 infection will be beneficial for virus therapy. Besides pulmonary injury, SARS-CoV-2 also causes cardiac injury, but the exact mechanisms are unclear. This review summarizes the essential structural characteristics of SARS-CoV-2 and angiotensin-converting enzyme 2 (ACE2), describes the cardiac manifestations following SARS-CoV-2 infection, and explores the mechanisms of cardiac injury targeting ACE2 after the viral invasion. We aim to help the timely detection of related symptoms and implementation of therapeutic measures by clinicians for SARS-CoV-2 infection.

## 1. Introduction

In December 2019, pneumonia outbreaks with unexpected results occurred throughout the world. Scientists subsequently identified a novel coronavirus, which lifted the veil of this infectious viral pneumonia [1,2]. This novel coronavirus pneumonia caused widespread concern throughout the world. In February 2020, the International Committee on Taxonomy of Viruses gave this coronavirus an official name, “SARS-CoV-2.” At the same time, the World Health Organization (WHO) named it “coronavirus disease 2019 (COVID-19)” [3,4]. This epidemic has now spread to all parts of the world. As of 18 January 2021, the confirmed total number of COVID-19 cases reached 93,805,612 globally, and the total number of deaths was 2,026,093 [5]. These figures continue to rise each day and bring a serious threat to human health, social and economic development, and the global medical and public health system. Scientists around the world are actively developing effective treatment strategies, focusing on vaccines and antiviral agents. The most frequently used antiviral agents include chloroquine, hydroxychloroquine, and remdesivir. Among them, the more in-depth studies are focusing on remdesivir.

Remdesivir belongs to inhibitors of viral RNA polymerase/RNA synthesis; it has a broad spectrum antiviral activity against several viruses [6,7,8]. Replication of SARS-CoV-2 requires the viral RNA-dependent RNA polymerase (RdRp) enzyme, a target of remdesivir. It has shown in vitro activity against SARS-CoV-2. Remdesivir appears to have a favorable clinical safety profile [9]. SARS-CoV-2 can enter the body through glycoprotein recognition, and this process can be blocked by vaccines. When the vaccines act on the body, they can induce the body to produce neutralizing antibodies targeting glycoprotein and block the virus from entering the host cells. Previously, a variety of vaccines had been designed based on SARS-CoV and MERS-CoV, among which the vaccines entering clinical trials included inactivated virus vaccine, nucleic acid vaccine, and vector vaccine [10,11,12,13]. Recently, some SARS-CoV-2 vaccines have been developed, and their efficacy and safety have been preliminarily proved. These vaccines mainly include nucleic acid vaccines BNT162b2 mRNA and mRNA-1273 [14,15]. Collectively, safe and effective vaccines and antiviral drugs are the most effective measure to curb the spread of the virus. Therefore, further exploration of the molecular mechanism of SARS-CoV-2 infection can better deal with its infection risk.

SARS-CoV-2, SARS-CoV, and Middle East respiratory syndrome coronavirus (MERS-CoV) belong to the *Betacoronavirus* genus, which are positive single-stranded RNA viruses that can infect a large number of mammals, including humans [16]. Research is currently ongoing concerning SARS-CoV-2 pathogenic mechanisms. More specifically, studies on angiotensin-converting enzyme 2 (ACE2) as an invasion target have received widespread attention. Considering the degree of high homology in gene sequences of SARS-CoV and SARS-CoV-2 [17], researchers have used computer-guided homology modeling to confirm further that the spike (S) protein amino acid sequences are 76.5% homologous between the two viruses, which also share an almost identical three-dimensional structure in their receptor-binding domain (RBD) and maintain similar van der Waals forces and electrostatic relationships in their interactions [18]. Previous investigations illustrate that the spike protein has a high binding affinity for ACE2 [19]. Recently, scientists have also found that through the mediation of the S protein, SARS-CoV-2 can invade host cells using ACE2 as the target [2,20]. ACE2 is an essential enzyme in the renin-angiotensin system (RAS), effectively maintaining RAS equilibrium [21]. RAS balance is widely recognized as critical to maintaining normal heart function. Researchers have recently employed single-cell RNA sequencing technology to analyze ACE2 expression in various human organs and cells. They found that ACE2 is not only highly expressed in type II alveolar epithelial cells and lower respiratory tract mucosal epithelial cells but also in the myocardium, vascular endothelial cells, ileal and esophageal epithelium, proximal renal tubules, and bladder epithelial cells, suggesting that the heart is also a high-risk target for SARS-CoV-2 infection [22]. From clinical practice, 29.3–45.7% of COVID-19 patients have associated cardiac injury, which is closely related to the case fatality rate [23,24,25]. The underlying mechanism for cardiac injury may be related to ACE2 depletion caused by direct SARS-CoV-2 binding with ACE2, thus leading to RAS imbalance. It might also be related to the indirect triggering of cytokine storm, yet the specific underlying mechanisms are currently unknown [26,27].

Here we summarize the structural characteristics and pathogenic mechanisms regarding SARS-CoV-2, focusing on the mechanism of cardiac injury targeting ACE2. We aim to aid clinicians with the timely detection of related symptoms and the effective implementation of therapeutic measures concerning SARS-CoV-2 infection.

## 2. The Basic Structure of SARS-CoV-2

**Figure 1:**
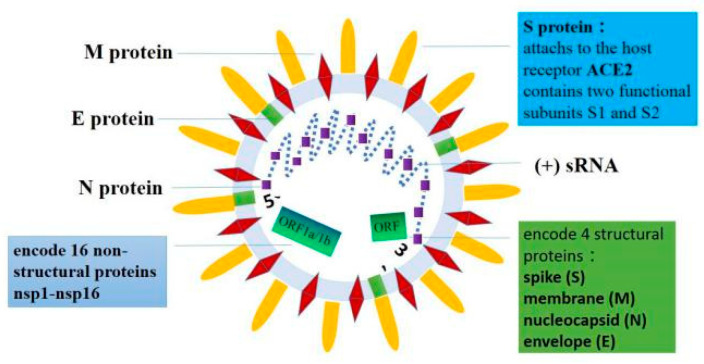
SARS-CoV-2 is a single-stranded RNA virus. Its specific genome composition includes untranslated regions (UTR) at both ends of the RNA strand and a complete open reading frame (ORF). The RNA strand of the SARS-CoV-2 genome has a methylated “cap” at the 5′ end, and a poly-A “tail” structure at the 3′ end. ORF1a and ORF1b at the 5′ end can encode 16 nonstructural proteins. The four ORFs at the 3′ end encode four structural proteins, namely, the spike (S), the envelope (E), the membrane (M), and the nucleocapsid (N) proteins. The S protein consists of two functional subunits, S1 and S2.

On 10 January 2020, the SARS-CoV-2 full genome sequence was published after metagenomic RNA sequencing. SARS-CoV-2 is a single-stranded RNA virus with a total genome length of approximately 29,903 nucleotides and constituting ten genes. Its specific genome composition includes untranslated regions (UTR) at both ends of the RNA strand and a complete open reading frame (ORF) that can encode 9860 amino acids [28,29]. The RNA strand of the SARS-CoV-2 genome includes a methylated “cap” at the 5′ end and a poly-A “tail” structure at the 3′ end. ORFs at the 5′ end can encode a series of viral replicases, of which ORF1b and ORF1a encode 16 nonstructural proteins. The four ORFs at the 3′ end encode four structural proteins, which are the spike (S), the membrane (M), the nucleocapsid (N), and the envelope (E) proteins, respectively [30,31]. The S protein functions critically in mediating the virus’s adsorption and fusion with the host cell membrane [31,32]. Wrapp et al. employed cryo-electron microscopy and 3D reconstruction techniques to obtain the S protein’s trimeric structure at a resolution of 3.5 Å [33,34]. The S protein belongs to trimeric class I fusion protein, which contains two functional subunits, S1 and S2. S1 binds to host cell receptors via its RBD, while S2 fuses the viral membrane with the host cell membrane, which allows the viral genome to enter the host cell. This entrance is an extremely complicated procedure that requires the synergy of receptor binding and proteolysis. The basic structure of SARS-CoV-2 forms the structural foundation for cardiac injury via the targeting of ACE2 during the invasion. The basic structure of SARS-CoV-2 is summarized in Figure 1.

## 3. The Basic Structure and Physiological Role of ACE2

On ACE2 is a metalloproteinase with a total length of 805 amino acids, including a 17-amino-acid N-terminal signal peptide (one zinc-binding motif) and a C-terminal membrane anchor [35,36]. A unique collectrin domain is also included. Although ACE2 and ACE are homologs and are both parts of the RAS, their actions are opposite [37]. ACE can convert angiotensin (Ang) I into Ang II, which can activate the G protein-coupled receptor angiotensin II type 1 receptor (AT1R) to promote vasoconstriction, increase vascular permeability, and mediate inflammatory responses. ACE2 can cleave Ang I and produce Ang-(1-9) peptide that can be transformed into vasodilator peptide Ang-(1-7) through ACE and other peptidases. In addition, ACE2 can hydrolyze Ang II into Ang-(1-7). In turn, the latter can act on Mas receptors to achieve vasodilation; reduce vascular permeability; exert antiproliferative, antioxidant, and antithrombotic effects; and reverse myocardial hypertrophy, left ventricular remodeling, and myocardial fibrosis. Therefore, it exerts essential functions in the cardiovascular system [38,39,40]. In the human body’s normal physiological state, the ACE2/Ang-(1-7)/MAS and ACE/Ang II/AT1R axes are in a state of dynamic equilibrium, thereby maintaining the normal functioning of the heart.

The cardiovascular system appears to have complex interactions with COVID-19. Poor clinical outcomes are associated with COVID-19 patients who have hypertension, coronary heart disease, or cardiac injury [24,41]. A vicious circle between SARS-CoV-2 infection and cardiac dysfunction or heart failure may occur. The putative relationship between them may relate to the role of ACE2. ACE2 is a key element in the renin–angiotensin–aldosterone system (RAAS), which systemically affects the vasculature and blood pressure [42]. SARS-CoV-2 virus enters human cells via binding its surface “spike” to ACE2. This interaction was significantly enhanced in patients with hypertension, coronary heart disease, diabetes, or other comorbidities [43]. Within the heart, cardiomyocytes, endothelial, and pericytes all express ACE2 [44]. Since ACE2 plays an important role in SARS-CoV-2 infection, the high expression of ACE2 in the heart increases the risk of SARS-CoV-2 infection.

A cardio-protective effect of ACE2 has been reported in various animal models and clinical studies [45,46,47]. Previous studies have consistently shown that the function of ACE2 is lost when SARS-CoV-2 binds to ACE2, which is mainly due to endocytosis and activation of proteolysis [48]. ACE2 expression was significantly decreased and myocardial dysfunction occurred in mice after human SARS-CoV infection, indicating that ACE2 plays a key role in mediating SARS-CoV infection in the heart [49]. If ACE2 does protect heart function in SARS-CoV-2 patients, the loss of ACE2 will further aggravate the burden on the heart, which forms a vicious circle. A lot of evidence indicates that the cardiovascular system is involved in the severity of disease infected by COVID-19, but the specific potential mechanism is still unclear. Further clinical trials are needed to clarify the role of ACE2 in SARS-CoV-2 patients and the detailed molecular mechanism of cardiac injury and heart failure.

## 4. Structural Characteristics Causing Cardiac Injury

Similarities in the structures and gene sequences between the S proteins of SARS-CoV-2 and SARS-CoV suggest that they might share a receptor, i.e., the ACE2 [50]. Subsequent studies confirmed that similar to SARS-CoV, the SARS-CoV-2 S protein mediates host cell entry, with ACE2 as the invasion target [51,52,53,54,55]. Compared with SARS-CoV, the structural conformation regarding the SARS-CoV-2 S protein interaction with ACE2 has been preserved overall, despite some changes in four essential amino acids in five binding sites regarding the SARS-CoV-2 S protein to the human ACE2. The SARS-CoV-2 binding affinity is 10–20 times stronger than that of SARS-CoV, explaining the enhanced human-to-human SARS-CoV-2 transmission [20,56]. In addition, a study by Wan’s team further confirmed that residue 394 (glutamine) of the RBD of the SARS-CoV-2 S protein corresponds to residue 479 in SARS-CoV, which could be identified via the key lysine 31 residue of the human ACE2 receptor. This finding suggests that SARS-CoV-2 may recognize human ACE2 more effectively compared with SARS-CoV [57].

Furthermore, ACE2 exists as a dimer in the human body and exhibits both open and closed conformations. These two conformations contain mutual recognition sites for coronaviruses, thus providing SARS-CoV-2 favorable conditions to infect humans with ACE2 as the target. The distribution of ACE2 in the human body is organ- and cell-specific. Using single-cell RNA sequencing, Zou et al. revealed that ACE2 is highly expressed in type II alveolar epithelial cells and cardiomyocytes, which indicates that the heart is also an important organ involved in SARS-CoV-2 infection [22].

## 5. The Possible Mechanism of Cardiac Injury following the Targeting of ACE2 after Viral Invasion

It has been reported that different levels of cardiac injury occurred in some hospitalized patients with COVID-19 [58,59]. However, the mechanism regarding cardiac injury due to SARS-CoV-2 infection is not entirely elucidated. Based on published literature and previous studies on SARS-CoV, two possible mechanisms are proposed: direct and indirect injury [60]. The direct injury mechanism refers to the cardiac injury caused by SARS-CoV-2 resulting from the targeting of ACE2 upon invasion. The indirect injury mechanism refers to the cardiac injury caused by cytokine storms following the host’s immune responses. Previous investigations show that cytokine storm after SARS-CoV-2 infection may also result from targeting ACE2 [61].

**Figure 2:**
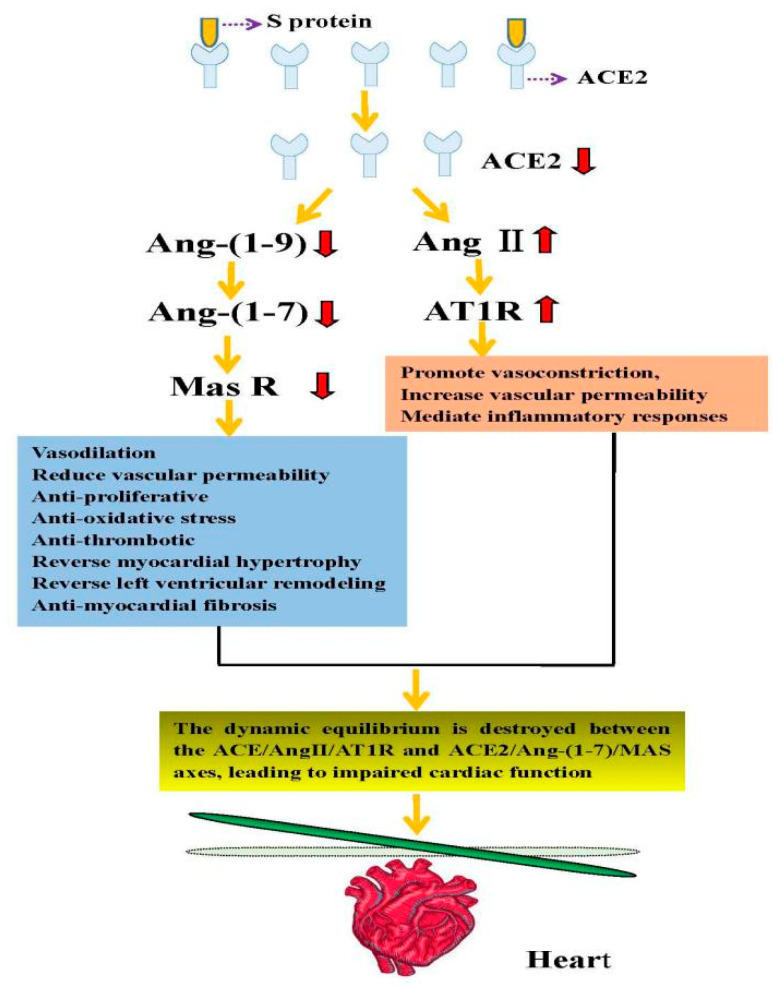
The direct mechanism of cardiac injury caused by SARS-CoV-2 using ACE2 as the target. Angiotensinogen is converted to Ang I by renin. Angiotensin-converting enzyme (ACE) can convert Ang I into Ang II, which in turn can activate the angiotensin II type 1 receptor (AT1R). ACE2 can cleave Ang I to produce the Ang-(1-9) peptide, which can then be converted into the vasodilator peptide Ang-(1-7) through ACE or other peptidases. Conversely, ACE2 can hydrolyze Ang II into Ang-(1-7), which acts on Mas receptors. When the SARS-CoV-2 S protein binds to ACE2 receptors, the dynamic equilibrium is destroyed between the ACE/Ang II/AT1R and ACE2/Ang-(1-7)/MAS axes, leading to impaired cardiac function.

Since SARS-CoV-2 virus enters human cells via binding its surface “spike” to ACE2, the high expression of ACE2 in the cardiovascular system may directly accelerate the attack of SARS-CoV-2 virus [44]. Some studies have reported that ACE2 can exert myocardial protective effects, reduce myocardial fibrosis, and improve cardiac function [38,39,40,62]. Oudit et al. conducted in vivo experiments on mice and found they can develop ACE2-dependent myocardial infection [49]. In addition, autopsies of SARS patients have also documented reductions in ACE2 mRNA and protein expression levels in cardiomyocytes, with macrophage infiltration and frank myocardial damage [49]. Moreover, the upregulation of ACE2 has been found after using RAAS inhibitors such as ACE inhibitors and angiotensin receptor type 1 blockers (ARBs) [63], which is more likely to increase the patient’s susceptibility to SARS-CoV-2 or show a worse prognosis [59,64]. To the contrary, other research revealed that it would not increase the risk of susceptibility [65,66,67]. Previous studies have shown that the function of ACE2 is lost when SARS-CoV-2 binds to ACE2 [48], which counteracts the cardioprotective effect of ACE2. Besides, this will create a vicious circle, aggravating heart damage. Further investigation revealed that the patient’s serum Ang II levels were significantly elevated. This finding may be because once the human body is infected with SARS-CoV-2, ACE2 will be depleted, which will affect the dynamic equilibrium between ACE2/Ang-(1-7)/MAS and ACE/Ang II/AT1R axes, eventually leading to impaired cardiac function. The direct injury mechanism of SARS-CoV-2 using ACE2 as a target, leading to cardiac injury, is summarized in Figure 2.

In addition, the indirect injury mechanism is also essential. Cytokine storm caused by immune disorder may be a key mediator. The hypothesis is that the overactivated immune system is one of the causes of SARS-CoV-2 induced heart injury. Sriramula et al. have shown that in rat models of Ang II-induced hypertension, a significant increase in inflammatory cytokines such as interleukin 6 (IL-6), interleukin 1β (IL-1β), and tumor necrosis factor α (TNF-α) occurs. In contrast, after the enhanced expression of ACE2, there was a decrease in the relevant inflammatory cytokines, and the hypertension was controlled [68]. Haga et al. found that after SARS-CoV infection there was a reduction in ACE2 and a significant rise in inflammatory cytokines, including TNF-α and IL-1β [69,70]. These two studies suggest that the release of inflammatory cytokines may be related to ACE2. In senescent mice that were infected with SARS-CoV, CD4+ T cells can produce neutralizing antibodies and balance the immune response [71]. Along with exhaustion of CD4+ and CD8+ T cells, severe immune system disorders appear in SARS-CoV-2 patients [72]. Another retrospective study of 69 COVID-19 patients found that patients showed elevated hs-TNI, CK-MB, IL-6, C-reactive protein, and procalcitonin, whereas their lymphocyte count and CD4+/CD8+ ratio were reduced [73]. In a case analysis published by Huang et al., COVID-19 patients showed elevated IL-1β, interferon γ (IFN-γ), monocyte chemoattractant protein 1 (MCP-1), and interferon-induced protein 10 (IP-10). Critically ill intensive care unit (ICU)patients showed more pronounced increases in IL-7, IL-2, IL-10, granulocyte-macrophage colony-stimulating factor (GM-CSF), MCP-1, IP-10, and TNFα compared with patients with mild disease. These findings indicate that cytokine storms are related to the severity of viral infection [58].

NLRP3 inflammasome, the powerful proinflammatory system, may contribute to the initiation of a cytokine storm in the development of SARS-CoV-2 pathologies. Proinflammatory stimuli such as cell debris can induce the expression of NLRP3 and other inflammatory body components in cardiomyocytes [74]. In fact, persistent virus shedding occurs in the death cases of COVID-19 infection. These virus fragments act heterologously to further activate the immune system and eventually lead to a cytokine storm. SARS-CoV-2 spike protein may directly trigger the activation of enzyme activity and downstream signal transduction after binding to ACE2 expressed on cell surface or release some effective cleavage fragments like C3a and C5a, which can directly trigger the activation of NLRP3 inflammatory bodies. Inflammation of the vascular system can lead to diffuse microvascular disease and thrombosis and then affect the function of the cardiovascular system.

In summary, SARS-CoV-2 infection is commonly followed by an ACE2 decrease, RAS imbalance, Ang II elevation, and a sharp increase in proinflammatory cytokines, thereby directly or indirectly damaging cardiomyocytes. Direct and indirect injury are not completely independent in clinic. Direct injury can lead to overactivation of the inflammatory response, which in turn aggravates the damage of the cardiovascular system.

## 6. Manifestations of Cardiac Injury

Researchers in China and abroad have performed autopsies and puncture histopathology on patients who died of SARS-CoV-2 infection. They found degeneration and necrosis in cardiomyocytes; a small amount of monocyte, lymphocyte, and (or) neutrophil infiltration in the interstitium; and endothelial cell shedding, intimal inflammation, and thrombosis in a portion of blood vessels [75]. In terms of clinical symptoms, Huang et al. were the first to publish a study involving 41 patients. Among the 41 COVID-19 patients in their study, five patients had acute myocardial injuries, manifesting elevated levels of serum hypersensitive troponin I (hs-cTHI) (>28 pg/mL), four of whom were critically ill and admitted to ICU for treatment [58]. Subsequently, another team analyzed 84 cases of COVID-19 and found that some patients showed an increase in their myocardial enzyme spectrum, especially in creatine kinase (CK) and creatine kinase-myocardial band (CK-MB), suggesting the presence of myocardial injury [59]. In a clinical analysis of COVID-19 diagnosed by healthcare workers, five of the 30 confirmed cases showed concomitant myocardial injury [60]. Furthermore, among the 138 COVID-19 cases confirmed and enrolled in Wang et al., ten patients presented cardiac injuries. Among them, ICU patients had significantly higher CK-MB and hs-cThI levels than non-ICU patients. Similarly, among these 138 confirmed cases of COVID-19, 23 cases showed arrhythmia, but further analysis could not be performed as the specific type of arrhythmia was unknown, which indicates the necessity of electrocardiogram monitoring in clinical work [76]. Furthermore, among the 99 confirmed cases of COVID-19 published by Wuhan Hospital in China, 11 patients died of sudden cardiac death, and these patients did not have a history of cardiovascular disease [77]. Peng et al. reported another 112 confirmed cases of COVID-19. Among them, 27.6% had a concomitant cardiovascular disease and a higher case fatality rate [78].

There are some phenotypes of cardiac injury induced by COVID-19 infection. Myocardial injury has been reported in 20–30% of hospitalized patients with COVID-19 infection [79]. When combined with basic diseases such as hypertension, diabetes, and coronary heart disease, the risk of death will increase. Microvascular and coagulation dysfunction are also manifestations of cardiac injury. Multiple reports confirmed that the elevated plasma D-dimer and fibrin degradation products that occurred were accompanied by prolonged prothrombin time or thrombocytopenia in COVID-19 infection patients [76,80]. Diffuse microvascular disease and thrombosis may lead to heart and other organ infarction and further worsen multiple organ failure. Arrhythmia is also a phenotype of heart injury. In a report of 138 hospitalized COVID-19 patients, arrhythmias occurred in 16.7% of patients [81]. Inflammation and cytokine storm may also reflect the manifestations of cardiac injury, it may be a phenotype or a cause of further aggravation of cardiac injury as discussed above.

In conclusion, SARS-CoV-2 infection is likely to result in cardiac injury, and the degree of cardiac injury is positively related to disease severity. Therefore, clinical symptoms should be closely observed to provide early treatment and reduce mortality (Table 1).

## 7. Therapeutic Strategies Using ACE2 as a Potential Target

There are currently no effective and specific therapeutic strategies for SARS-CoV-2 treatment. Many treatment options may be derived by summarizing the treatment experiences for SARS and MERS. The primary research and development (R&D) strategy is the screening of existing broad-spectrum antiviral drugs and further developing drugs that can simultaneously target the virus and the host [82,83,84,85,86]. Previous studies have found that SARS-CoV-2 may invade host cells via ACE2 receptors, which has provided many targets for the R&D of treatments and SARS-CoV-2 vaccines, including the blockade of the SARS-CoV-2 S protein binding with ACE2 receptors via the application of ACE inhibitors, among other strategies [87,88,89].

Several glycosylation sites can be found in the extracellular structure of ACE2 expressed in mammalian cells. The glycosylation of these sites may affect SARS-CoV-2 S protein binding with the ACE2 receptor, providing us with new strategies to block SARS-CoV-2 S protein binding with the ACE2 receptor [35]. Additionally, recent studies have discovered that chloroquine can block viral infection through the improvement of endosomal pH required for virus/cell fusion and interfere with ACE2 terminal glycosylation [54,85]. Furthermore, China has listed chloroquine phosphate as a therapeutic drug for COVID-19 patients in the “Diagnosis and Treatment Protocol for Coronavirus Disease 2019 (Trial Version 7)” and has included this drug in large-scale clinical trials [90]. Several medical teams in China and abroad have developed ACE2 antibodies, S protein antibodies, and others based on the blockade of binding between the ACE2 receptor and the SARS-CoV-2 S protein [87,88,89,91]. At present, there is still much controversy surrounding the use of ACE inhibitor-like drugs in patients with cardiac injury after SARS-CoV-2 infection. There are two main types of ACE inhibitor-like drugs that have received significant attention: angiotensin-converting enzyme inhibitors (ACEI) and angiotensin II receptor blockers (ARB). SARS-CoV-2 infection can lead to decreased ACE2, an imbalance between the ratio of ACE and ACE2, an absolute or relative increase in Ang II, and an over-activation of AT1R, which can result in impaired cardiac function. ACEI and ARB may inhibit the above pathophysiological changes and improve cardiac injury after SARS-CoV-2 infection [92,93,94].

Meanwhile, some reports revealed that the use of common classes of antihypertensive medications did not increase the risk of positive or severe COVID-19 [65,66,67]. These data support the continuation of existing treatment for patients with hypertension during the COVID-19 pandemic. There are also some contrary views: some studies revealed that although administering hypertensive rats with ACEI/ARB can reduce their blood pressure, their ACE2 levels were elevated by 4.7- and 2.8-fold, respectively. This means the application of ACEI/ARB may increase ACE2 negative feedback and increase infection risk [63]. Liu et al. argued that the application of ACE inhibitor-like drugs might increase cells’ susceptibility to viral invasion or worsen the disease [95]. This supposition is because ACE inhibitors can lead to an increase in bradykinin levels, which can lead to vasodilation and lower blood pressure, as well as causing edema and exacerbating the inflammatory response.

In summary, the application of ACE inhibitor-like drugs after SARS-CoV-2 infection, especially in patients with cardiac injury, still requires support from a large amount of clinical data to validate these conclusions.

## 8. Discussion

The epidemic caused by SARS-CoV-2 infection has achieved global focus. The WHO has also designated this viral epidemic as a significant global public health emergency. However, thus far, our understanding of SARS-CoV-2 is only the “tip of the iceberg.” Many researchers have reported that the SARS-CoV-2 sequence identity is at most 88% homologous with bat-derived coronaviruses, suggesting that bats may be a natural host [1,28]. Subsequently, researchers from the South China University of Technology found through the analysis of more than 1000 metagenomic samples that pangolins may also be a candidate intermediate SARS-CoV-2 host [96]. The primary sources of SARS-CoV-2 infection are persons infected with this virus. The main transmission routes are via respiratory droplets and contact transmission, with high population susceptibility [97,98]. Concerning SARS-CoV-2 infection pathogenesis, current studies were combined with the existing literature to conclude that SARS-CoV-2 invades host cells via the mediation of the S protein targeting ACE2. Many experimental studies and reports of clinical symptoms in patients with COVID-19 suggest that the heart is a potential target organ for SARS-CoV-2 infection. Moreover, the mechanism of cardiac injury may be caused directly by ACE2 depletion resulting from SARS-CoV-2 binding with ACE2 and indirectly by a cytokine storm. It is hoped that the findings of the above studies will provide new directions for the future development of ACE2 as a therapeutic target for cardiac injury after SARS-CoV-2 infection, as well as for related vaccines.

Based on a large number of reported clinical cases of SARS-CoV-2, we found that in addition to cardiac and pulmonary injury, SARS-CoV-2 infection might also lead to kidney, liver, gastrointestinal tract, testicular, and even ocular injury. This damage may be due to the organ and cell specificity of ACE2 distribution, such that SARS-CoV-2 can attack multiple organs using ACE2 as a target [22,99]. It may also be related to the immune defense mechanisms, whereby the intensity of the immune response varies from individual to individual, and some studies have shown that autoimmune attack may also cause multiple organ injuries. This response will be a key focus in future research [100]. The mechanism of cardiac injury after SARS-CoV-2 infection remains unclear. Apart from the mechanism involving ACE2 as the target of invasion, which is highlighted in this review, there may be many other mechanisms involved, such as the series of pathophysiological changes induced by hypoxemia, leading to cardiac injury. Disorders of pulmonary gas exchange in patients with COVID-19 may lead to hypoxemia, while acidosis, oxidative stress, and induced inflammatory reactions during hypoxia and reperfusion can exacerbate cardiac injury [75]. Finally, among patients infected with SARS-CoV-2, 50% have chronic underlying diseases, such as hypertension, heart disease, and diabetes. These patients are more prone to develop severe diseases. During the treatment of these patients, SARS-CoV-2 infection was only the initial illness, and the final causes of death are often heart failure and multiple organ dysfunction [25,101,102]. Therefore, we should be more vigilant when it comes to the cardiac condition of COVID-19 patients in clinical practice, complying with the principles of individualization in the application and selection of ACE inhibitor-like drugs for these patients.

Of course, safe and effective vaccines are also urgently needed. As we know, it is not feasible for COVID-19 to obtain group immunity through human infection. In the development process of new crown vaccines, researchers are facing a variety of problems and challenges, such as the weak immunogenicity of a single dose vaccine, incomplete virus inactivation, disease risk, and safe mass production. However, we believe that with the development of effective vaccines and antiviral drugs we will eventually defeat SARS-CoV-2 infection.

## Figures and Tables

**Table 1:** The pathological and clinical features of cardiac injury caused by SARS-CoV-2.

Pathological	Clinical Features
Cardiomyocytes: degeneration necrosis	Acute myocardial injury
Interstitium: monocyte, lymphocyte, and (or) neutrophil infiltration	Arrhythmia
Blood vessels: endothelial cell shedding intimal Inflammation thrombosis	Sudden cardiac death

## Data Availability

No new data were created or analyzed in this study. Data sharing is not applicable to this article.
